# IGF-1 supplementation improves *in vitro* maturation quality of *Capra hircus* oocytes via PDE3A activation, gap junction remodeling, apoptosis suppression, and kisspeptin signaling

**DOI:** 10.3389/fvets.2026.1821251

**Published:** 2026-05-29

**Authors:** Widjiati Widjiati, Epy Muhammad Luqman, Ninik Darsini, Aulanni’am Aulanni’am, Viski Fitri Hendrawan, Devia Yoanita Kurniawati, Wan Nor Fitri Bin Wan Jaafar

**Affiliations:** 1 Department of Veterinary Anatomy, Faculty of Veterinary Medicine, Universitas Airlangga, Surabaya, Indonesia; 2 Department of Medical Biology, Faculty of Medicine, Universitas Airlangga, Surabaya, Indonesia; 3 Department of Biochemistry, Faculty of Veterinary Medicine, Universitas Brawijaya, Malang, Indonesia; 4 Department of Veterinary Reproduction, Faculty of Veterinary Medicine, Universitas Brawijaya, Malang, Indonesia; 5 Doctoral Program of Veterinary Sciences, Faculty of Veterinary Medicine, Universitas Airlangga, Surabaya, Indonesia; 6 Department of Farm and Exotic Animal Medicine and Surgery, Faculty of Veterinary Medicine, Universiti Putra Malaysia, Serdang, Selangor, Malaysia

**Keywords:** apoptosis, gap junction, IGF-1, *in vitro* maturation, kisspeptin, oocyte, PDE3A, Zero Hunger

## Abstract

**Introduction:**

Although follicular development and oocyte competence depend on insulin-like growth factor-1 (IGF-1), its optimal dose and molecular mechanisms during goat *in vitro* maturation (IVM) remain unclear.

**Methods:**

Cumulus-oocyte complexes from *Capra hircus* were cultured for 22 h in IVM media containing 0, 50, 100, or 150 ng/mL IGF-1, and nuclear maturation was assessed by first polar body extrusion. To elucidate mechanisms, Gja4 and PDE3A mRNA, IL-6, caspase-3, and caspase-9 mRNA were measured by qPCR, kisspeptin protein was quantified by ELISA, and correlation and structural path analyses were performed.

**Results:**

IGF-1 at 100 ng/mL significantly increased maturation rate compared with control, whereas 150 ng/mL provided no additional benefit, indicating a biphasic response. At 100 ng/mL, PDE3A expression and kisspeptin protein levels increased, while Gja4 and caspase-3 expression decreased; IL-6 and caspase-9 remained unchanged. Correlation and structural path analyses identified PDE3A-mediated meiotic restart and suppression of caspase-3-dependent apoptosis as primary drivers of improved maturation.

**Discussion:**

Optimized IGF-1 supplementation enhances goat oocyte quality through coordinated regulation of meiotic activation, gap junction remodeling, and apoptosis inhibition. These findings may improve the efficiency of goat in vitro embryo production and support Sustainable Development Goal 2 (Zero Hunger) by strengthening reproductive efficiency and livestock productivity.

## 1 Introduction

The domestic goat (*Capra hircus*) plays an important role in livelihoods and food security in many tropical developing countries. In Indonesia, indigenous goat genetic resources contribute substantially to rural economies and provide essential animal-source foods, including meat and milk, while remaining well adapted to tropical environments (1, 2). However, productivity in local goat populations often remains below genetic potential due to multifactorial constraints, including reproductive limitations, genetic background, and management practices (3). In practice, abattoir-derived ovaries are widely used as a key source of oocytes for research and *in vitro* production (IVP) programs. Yet, because females entering slaughter may include animals culled for reproductive problems, advanced age, or reduced performance, oocytes retrieved from these ovaries can show variable and sometimes reduced developmental competence.

Assisted reproductive technologies (ART) offer opportunities to accelerate genetic improvement and safeguard valuable livestock germplasm. *In vitro* embryo production (IVP) is a cornerstone ART pipeline that includes oocyte recovery, *in vitro* maturation (IVM), in vitro fertilization (IVF), and embryo culture (4, 5). The overall efficiency of IVP depends strongly on oocyte quality and the capacity to complete nuclear and cytoplasmic maturation during IVM. Oocyte maturation involves meiotic resumption and acquisition of developmental competence, which requires coordinated regulation of cumulus–oocyte communication, intracellular signaling, and cellular survival pathways (6, 7).

A practical strategy to improve maturation outcomes is supplementation of maturation media with growth factors to better mimic the follicular microenvironment. Insulin-like growth factor-1 (IGF-1) is a pleiotropic peptide implicated in folliculogenesis and oocyte competence, supporting cellular growth, differentiation, and survival through IGF-1 receptor signaling and downstream pathways such as PI3K/Akt (8, 9). Across mammalian species, IGF-1 has been reported to promote meiotic resumption, support cytoplasmic maturation, and improve subsequent developmental outcomes, including studies in cattle, pigs, and goats (10–12). However, the optimal IGF-1 dose and the integrated molecular mechanisms driving improved goat IVM outcomes remain incompletely understood.

Mechanistically, several interrelated pathways are relevant. Gap junction communication between cumulus cells and the oocyte—mediated by connexins such as GJA4 (connexin 37)—maintains metabolic coupling and regulates the transfer of small molecules necessary for early maturation (13). Dynamic remodeling and/or closure of gap junctions is considered a physiological feature of meiotic progression, as persistent transfer of inhibitory signals can maintain meiotic arrest. In parallel, phosphodiesterase 3A (PDE3A) regulates intra-oocyte cyclic AMP (cAMP) levels; PDE3A-mediated cAMP hydrolysis is critical for releasing meiotic arrest and enabling progression to metaphase II (14, 15). Beyond maturation signaling, oocyte competence is also influenced by stress and apoptosis within the cumulus–oocyte complex. Apoptotic markers such as caspase-3 and caspase-9 and inflammatory mediators such as IL-6 can reflect cellular damage and reduced developmental competence during IVM (16, 17). In addition, kisspeptin signaling—classically recognized for regulating hypothalamic GnRH release—has gained attention for potential local roles in the ovary, including effects on follicular function and oocyte maturation (18, 19). Together, these pathways provide a framework to evaluate how IGF-1 supplementation may coordinate meiotic activation, intercellular communication, and apoptosis-related processes to improve oocyte maturation quality.

Therefore, this study aimed to (1) determine the optimal IGF-1 supplementation dose to improve IVM maturation rates in *Capra hircus* oocytes, (2) evaluate maturation-related markers (Gja4 and PDE3A), (3) assess apoptosis- and inflammation-related markers (caspase-3, caspase-9, and IL-6), (4) quantify kisspeptin levels, and (5) examine interrelationships among these variables using correlation and structural path analyses. Importantly, optimizing IVM conditions for small ruminants supports Sustainable Development Goal 2 (Zero Hunger) by strengthening reproductive efficiency and genetic improvement in livestock systems, which can increase the availability and stability of animal-source foods, improve productivity of local breeds, and enhance resilience of smallholder production in resource-limited settings through scalable ART and germplasm conservation strategies.

## 2 Materials and methods

### 2.1 Ethical clearance

All procedures involving animal-derived biological materials were approved by the Animal Care and Use Committee, Faculty of Veterinary Medicine, Universitas Airlangga, Surabaya, Indonesia (Approval No. 1. KEH.025.02.2024; issued February 25, 2024). The study followed institutional biosafety protocols and the ARRIVE 2.0 (Animal Research: Reporting of *In Vivo* Experiments) reporting guidelines. No live animals were sacrificed specifically for this research; ovaries were obtained from animals slaughtered for commercial meat production. Procedures complied with Indonesian animal welfare regulations, including Law No. 18/2009 and Government Regulation No. 95/2012 concerning livestock and animal health.

### 2.2 Study design and experimental timeline

**Figure 1:**
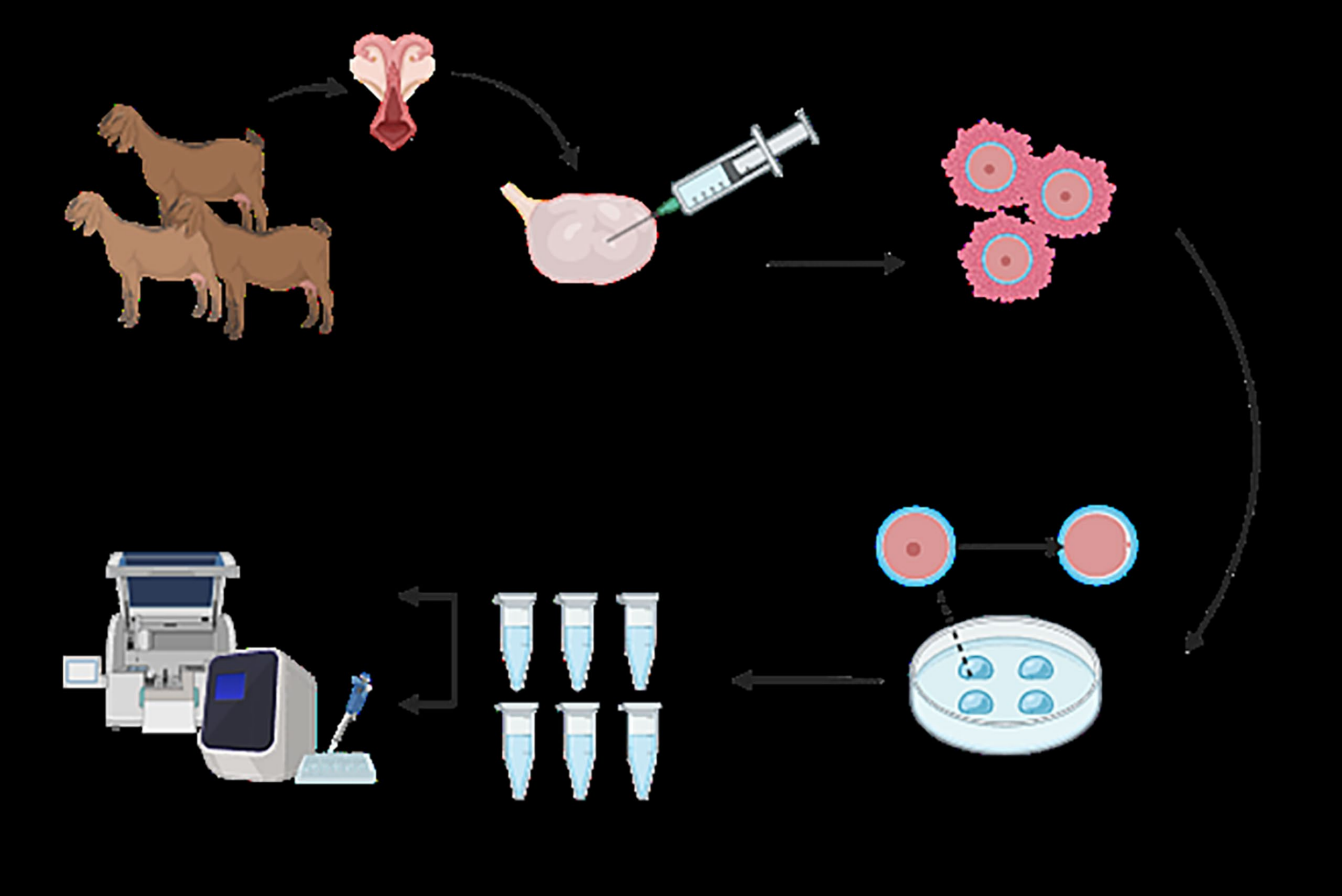
Summary of the study methodology. Ovaries were collected from a commercial abattoir and cumulus–oocyte complexes (COCs) were aspirated from ovarian follicles. COCs were matured *in vitro* in four IGF-1 concentrations (0, 50, 100, and 150 ng/mL) for 22 h, followed by assessment of nuclear maturation to metaphase II (MII) based on first polar body extrusion. The optimal IGF-1 dose (100 ng/mL) was subsequently used for molecular analyses, including qPCR assessment of maturation-related genes (*Gja4*, *PDE3A*) and apoptosis/inflammation-related genes (*IL-6*, *Casp3*, *Casp9*), and ELISA quantification of kisspeptin protein levels (Created with BioRender.com).

This experimental study was conducted from January to August 2025 and comprised three sequential phases: (1) ovary collection and retrieval of cumulus–oocyte complexes (COCs) from slaughterhouse specimens; (2) dose–response evaluation of IGF-1 supplementation during *in vitro* maturation (IVM) to determine the optimal concentration based on nuclear maturation rate; and (3) molecular analyses comparing the control group (0 ng/mL IGF-1) and the optimal IGF-1 group (100 ng/mL). A schematic overview of the experimental workflow is provided in Figure 1.

### 2.3 Ovarian collection and transportation

A total of 120 ovaries were collected from healthy, non-pregnant adult female Kacang goats (aged 6–12 months) immediately after slaughter at a commercial abattoir. While the estrous cycle stage was not determined, oocytes were selected based on strict morphological criteria to ensure a uniform starting population, though this remains a recognized limitation of abattoir-derived material. Ovaries were collected between 05:00 and 06:00 and transported to the laboratory within 1 h in insulated thermal flasks (Akebonno, Jakarta, Indonesia) containing warm (37–38 °C) 0.9% normal saline supplemented with streptomycin. Upon arrival, ovaries were rinsed three times with sterile phosphate-buffered saline (PBS) pre-warmed to 37 °C to remove blood and debris.

### 2.4 Oocyte recovery and selection criteria

To mitigate variability from unknown estrous stages, only follicles with a diameter of 6–8 mm were aspirated, and COCs were strictly selected based on the presence of three or more compact cumulus layers. Follicles were aspirated using a 10 mL syringe and an 18-gauge needle (Onemed, PT Jayamas Medical Industry Tbk, Sidoarjo, Indonesia) containing 1 mL sterile PBS. The follicular aspirates were examined under an Olympus X41 inverted microscope (Evident Scientific, Tokyo, Japan) to identify COCs. Selection was based on morphological criteria: only oocytes surrounded by three or more compact layers of cumulus cells and exhibiting homogeneous, evenly granulated cytoplasm were included. Oocytes showing incomplete cumulus investment, partial or total denudation, or signs of atresia (e.g., cytoplasmic darkening or vacuolization) were excluded. Experiments were performed in three independent biological runs on different days using freshly collected ovaries.

### 2.5 *In vitro* maturation and experimental groups

Selected COCs were washed serially and cultured in TCM-199 medium (Thermo Fisher Scientific, Waltham, MA, United States) supplemented with 10% fetal bovine serum (FBS; Cat. No. 12103C; Sigma-Aldrich, St. Louis, MO, United States), 10 μg/mL follicle-stimulating hormone (FSH), 10 μg/mL luteinizing hormone (LH), and 1% penicillin–streptomycin. Oocytes were randomly allocated to four experimental groups based on IGF-1 concentration:

- Control: 0 ng/mL IGF-1.
- T1: 50 ng/mL IGF-1.
- T2: 100 ng/mL IGF-1.
- T3: 150 ng/mL IGF-1.

For each group, 10–15 oocytes were placed in 30 μL microdroplets of maturation medium overlaid with mineral oil and incubated for 22 h at 38.5 °C in a humidified atmosphere containing 5% CO₂ (relative humidity >95%). The IGF-1 concentrations were selected based on previous studies indicating dose-dependent (biphasic) effects of IGF-1 on follicular and oocyte physiology in domestic species.

### 2.6 Assessment of nuclear maturation and selection of optimal IGF-1 concentration

After 22 h of IVM, nuclear maturation was assessed by identifying first polar body extrusion under an inverted microscope, indicating progression to metaphase II (MII). The maturation rate for each group was calculated as the percentage of MII oocytes relative to the total number cultured. Based on the dose–response outcomes, the concentration yielding the highest maturation rate (100 ng/mL) was selected as the optimal dose for subsequent molecular analyses. Molecular endpoints were subsequently compared between the control group (0 ng/mL) and the optimal IGF-1 group (100 ng/mL).

### 2.7 RNA extraction and cDNA synthesis

For gene expression analyses, each experimental group (control and 100 ng/mL IGF-1) included eight biological replicates, with each replicate consisting of 300 pooled matured oocytes stored in sterile cryotubes. Prior to RNA extraction, oocytes were processed together with surrounding cumulus cells or intact COCs. Total RNA was extracted using the Trizol Plus RNA Purification Kit (Invitrogen, Barcelona, Spain) in combination with the RNeasy Micro Kit (Qiagen, Hilden, Germany), following the manufacturers’ protocols. Briefly, oocytes were lysed in Buffer RZ, incubated at room temperature for 5 min, and centrifuged at 12,000 rpm for 5 min at 4 °C to remove insoluble components. The supernatant was transferred to fresh tubes, mixed vigorously with chloroform, and centrifuged at 12,000 rpm for 15 min at 4 °C to separate phases. The aqueous phase was collected, mixed with 70% ethanol, and loaded onto RNase-free spin columns. Columns were washed sequentially with Buffer RD and Buffer RW, followed by an additional dry spin for 2 min to remove residual ethanol. RNA was eluted in 20 μL RNase-free water.

RNA concentration was quantified using a Qubit fluorometer (Thermo Fisher Scientific, Waltham, MA, United States) with the Qubit RNA HS Assay Kit and stored at −70 °C until processing. First-strand cDNA was synthesized from 500 ng total RNA using the iScript cDNA Synthesis Kit (Bio-Rad Laboratories, Barcelona, Spain) in a 20 μL reaction containing 4 μL 5 × reaction mix, 1 μL reverse transcriptase, RNA template, and nuclease-free water. Thermal cycling conditions were: 25 °C for 5 min (priming), 46 °C for 20 min (reverse transcription), and 95 °C for 1 min (enzyme inactivation). Synthesized cDNA was stored at −20 °C until qPCR.

### 2.8 Quantitative real-time PCR

Quantitative real-time PCR was used to quantify mRNA expression of maturation-related genes (Gja4 and PDE3A) and apoptosis/inflammation-related genes (IL-6, caspase-3, and caspase-9). Reactions were run in duplicate using a LightCycler 480 II system (Roche, Barcelona, Spain) on 96-well plates. Each 20 μL reaction contained 2 μL cDNA template, 10 μL 1 × Power SYBR Green PCR Master Mix (Applied Biosystems, Foster City, CA, United States), 0.5 mM forward and reverse primers, and nuclease-free water to volume. Primer specificity and amplification efficiency were verified before analysis. Cycling conditions were: UDG activation at 50 °C for 2 min, initial denaturation at 95 °C for 3 min, followed by 40 cycles of 95 °C for 5 s and 55 °C for 30 s. Melt-curve analysis was performed from 65 °C to 95 °C with continuous fluorescence acquisition to confirm single-product amplification and absence of primer-dimer artifacts.

ACTB (*β*-actin) was used as the endogenous reference gene. Cycle threshold (Ct) values were obtained using LightCycler 480 software. Relative gene expression was calculated using the 2^−ΔΔCt method, where ΔCt = Ct_target − Ct_ACTB for each sample, and ΔΔCt = ΔCt_treatment − ΔCt_control. Results are reported as fold-change relative to control (mean ± SEM). Primer sequences, product sizes, and accession numbers are provided in Table 1.

**Table 1:** Primer sequences used for quantitative real-time PCR.

Target gene	Primer sequence (5′ → 3′)	Product size (bp)	Accession number
Gja4	F: CTGGCTGATCGTGGTGATCT	142	NM_001289153.1
	R: AAGGACACCAGGATGACGAA		
PDE3A	F: AGCACCTTCATCCAGTGCTA	153	XM_005680800.3
	R: TTCACAAGGGAACCTGGTCG		
IL-6	F: TCCAGAACGAGTATGAGGCA	134	NM_001285640.1
	R: CATCCGAATAGCTCTCAGGC		
Caspase 3	F: GGTTCATCCAGTCCCTTTG	148	NM_001286977.1
	R: AACCAGGTGCTGTAGAGTCC		
Caspase 9	F: GGCTGTTAAAGGCTATCGGA	151	XM_018060038.1
	R: CAGAGACGGTGTTCAGCATT		
*β*-actin	F: ACCACTGGCATTGTCATGGA	113	NM_001314342.1
	R: TCCTTGATGTCACGGACGAT		

Primer sequences (5′ → 3′), product size (bp), and accession numbers for Gja4, PDE3A, IL-6, caspase-3, caspase-9, and *β*-actin (ACTB).

### 2.9 Kisspeptin protein quantification by ELISA

Kisspeptin protein concentrations were measured using ELISA. Eight biological replicates were prepared per group (control and 100 ng/mL IGF-1), with each replicate consisting of 20 pooled oocytes. Oocytes were lysed in ice-cold PBS containing a protease inhibitor cocktail (Sigma-Aldrich) by sonication on ice. Lysates were centrifuged at 10,000 × g for 10 min at 4 °C, and supernatants were collected. Kisspeptin levels were quantified using a commercial ELISA kit (Cat. No. E-EL-R2530; Elabscience, Wuhan, China) according to the manufacturer’s instructions. Briefly, 100 μL of standards or samples were added to pre-coated 96-well plates and incubated for 90 min at 37 °C. Plates were washed, incubated with biotinylated detection antibody for 60 min, followed by horseradish peroxidase–conjugated streptavidin for 30 min. The reaction was developed using tetramethylbenzidine substrate and stopped with stop solution. Absorbance was read at 450 nm using a microplate reader (Bio-Rad, Hercules, CA, United States). Kisspeptin concentrations were calculated from the standard curve and expressed as pg/mL.

### 2.10 Statistical analysis

Statistical analyses were performed using SPSS Statistics version 2023 (IBM Corp., Armonk, NY, United States). Statistical significance was set at *p* < 0.05. Maturation rates among four IGF-1 concentrations (0, 50, 100, and 150 ng/mL) were compared using one-way analysis of variance (ANOVA) followed by Duncan’s multiple range post-hoc test. For molecular endpoints (qPCR and ELISA), differences between the control group and the optimal IGF-1 group (100 ng/mL) were evaluated using an independent samples *t*-test. The biological replicate (pooled oocyte sample) was used as the unit of analysis. Normality and homogeneity of variance were assessed using Shapiro–Wilk and Levene’s tests, respectively. Data are presented as mean ± standard error of the mean (SEM).

## 3 Results

### 3.1 Effect of IGF-1 supplementation on oocyte maturation rate

**Figure 2:**
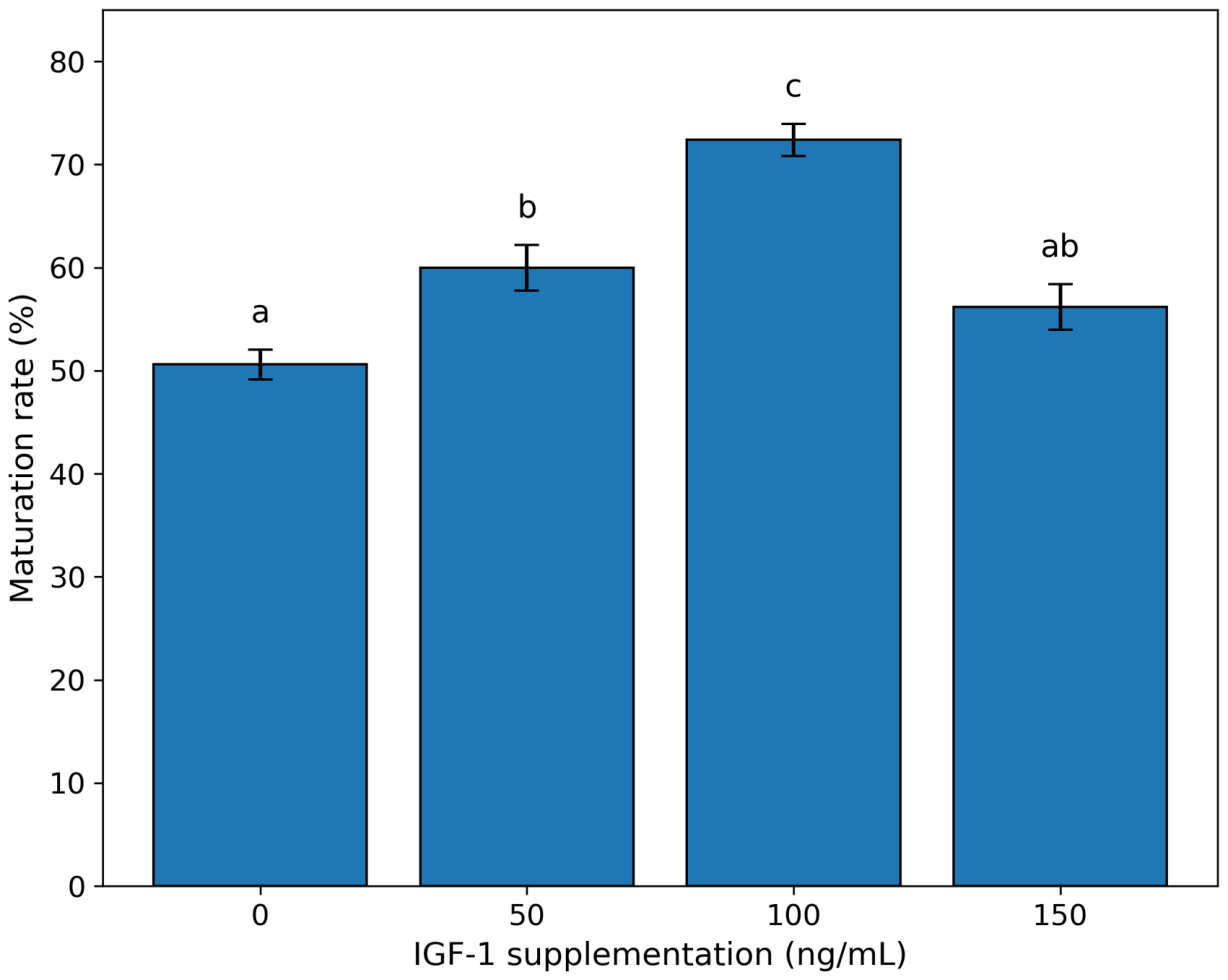
Dose-dependent effect of IGF-1 on oocyte maturation rate. Maturation rate (%) of *Capra hircus* oocytes after IVM with IGF-1 at 0, 50, 100, and 150 ng/mL. Bars represent mean ± SEM. Different letters indicate significant differences among groups (one-way ANOVA followed by Duncan’s multiple range test; *p* < 0.05).

IGF-1 supplementation significantly affected the nuclear maturation rate of *Capra hircus* oocytes (Table 2; Figure 2). The highest maturation rate was observed at 100 ng/mL (72.4 ± 1.56%), which was significantly higher than the control (0 ng/mL; 50.6 ± 1.46%) and other treatment groups (*p* < 0.05). Supplementation at 50 ng/mL increased maturation compared with control (60.0 ± 2.21%), but remained significantly lower than the 100 ng/mL group (*p* < 0.05). In contrast, the 150 ng/mL group (56.2 ± 2.21%) did not improve maturation relative to 50 ng/mL and was markedly lower than the 100 ng/mL group (*p* < 0.05), indicating a biphasic dose-dependent response. Based on these outcomes, 100 ng/mL was selected as the optimal concentration for subsequent molecular analyses.

**Table 2:** Effect of IGF-1 supplementation on oocyte maturation rate.

IGF-1 supplementation dose (ng/mL)	Maturation rates (%)
0	50.6^a^ ± 1.46
50	60^b^ ± 2.21
100	72.4^c^ ± 1.56
150	56.2^ab^ ± 2.21

Maturation rates (%) of *Capra hircus* oocytes cultured with IGF-1 at 0, 50, 100, and 150 ng/mL. Data are presented as mean ± SEM. Different superscript letters within the column indicate significant differences among groups (one-way ANOVA followed by Duncan’s multiple range test; *p* < 0.05).

### 3.2 Oocyte maturation–associated markers (Gja4, PDE3A) and kisspeptin protein levels

**Figure 3:**
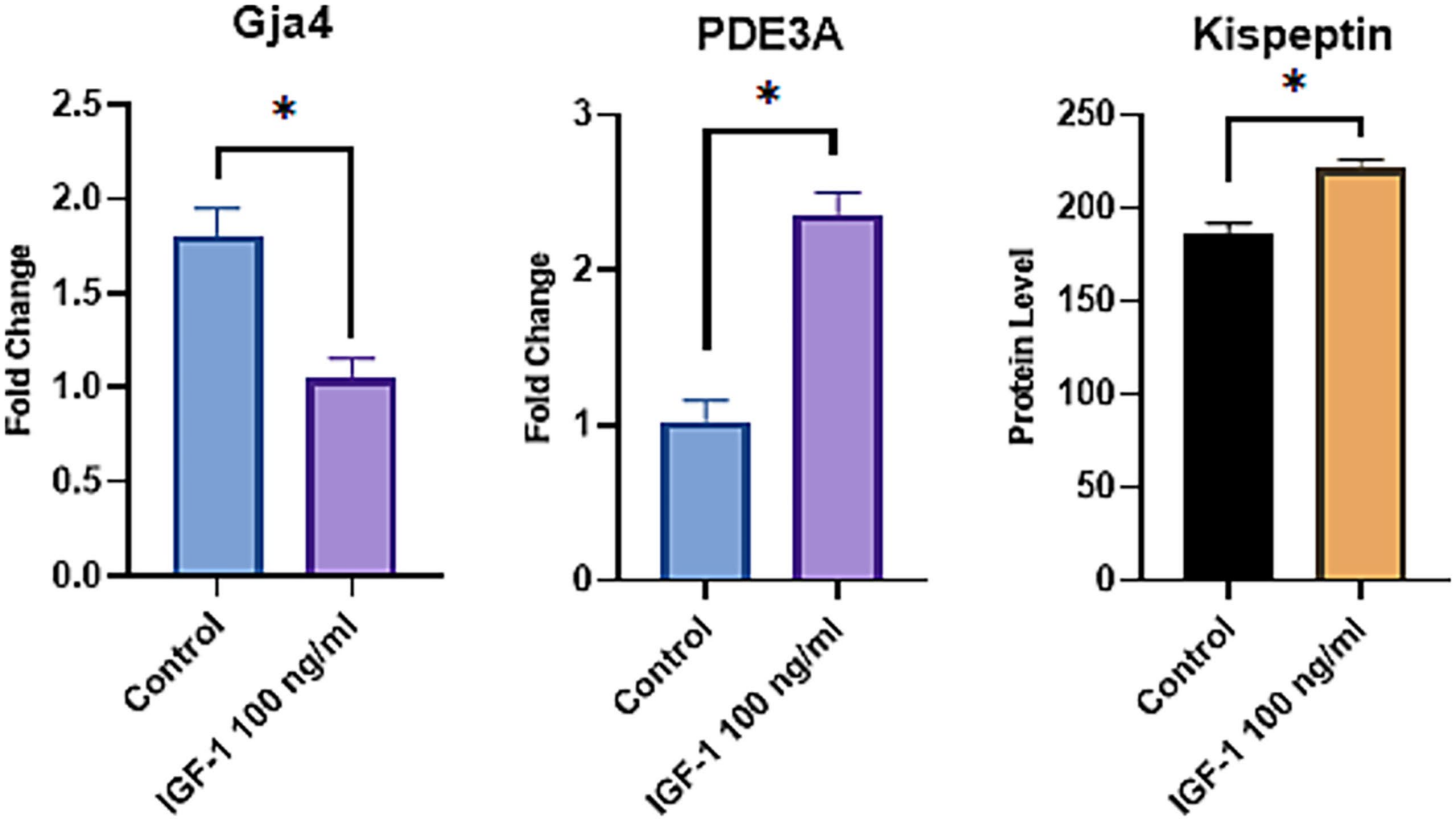
Effect of the optimal IGF-1 dose on maturation-related markers and kisspeptin levels. Relative mRNA expression of Gja4 and PDE3A and kisspeptin protein levels in control (0 ng/mL) and IGF-1 (100 ng/mL) groups. Data are presented as mean ± SEM (*n* = 8 biological replicates). Asterisks indicate significant differences between groups (independent samples *t*-test; *p* < 0.05).

Molecular analysis comparing the control group and the optimal IGF-1 group (100 ng/mL) showed significant modulation of maturation-associated markers (Table 3; Figure 3). Gja4 expression was significantly reduced in the IGF-1 group (1.05 ± 0.10) compared with control (1.81 ± 0.13) (*p* < 0.05). Conversely, PDE3A expression was significantly increased in the IGF-1 group (2.36 ± 0.13) compared with control (1.03 ± 0.12) (*p* < 0.05). In parallel, kisspeptin protein levels were significantly elevated following IGF-1 supplementation (221.47 ± 4.39 pg/mL) compared with control (186.79 ± 4.92 pg/mL) (*p* < 0.05).

**Table 3:** Effect of the optimal IGF-1 dose on maturation-related gene expression and kisspeptin protein levels.

Treatment Groups	*n*	mRNA relative expression (Mean ± SEM)		Protein level (Mean ± SEM)
		Gja4	PDE3A	Kisspeptin
Control	8	1.81^a^ ± 0.13	1.03^a^ ± 0.12	186.79^a^ ± 4.92
IGF-1 100 ng/mL	8	1.05^b^ ± 0.10	2.36^b^ ± 0.13	221.47^b^ ± 4.39

Relative mRNA expression of Gja4 and PDE3A and kisspeptin protein levels in control (0 ng/mL) and IGF-1 (100 ng/mL) groups. Data are presented as mean ± SEM; *n* = 8 biological replicates. Different superscripts within the same column indicate significant differences between groups (independent samples *t*-test; *p* < 0.05).

### 3.3 Apoptosis- and inflammation-associated markers (IL-6, caspase-3, caspase-9)

**Figure 4:**
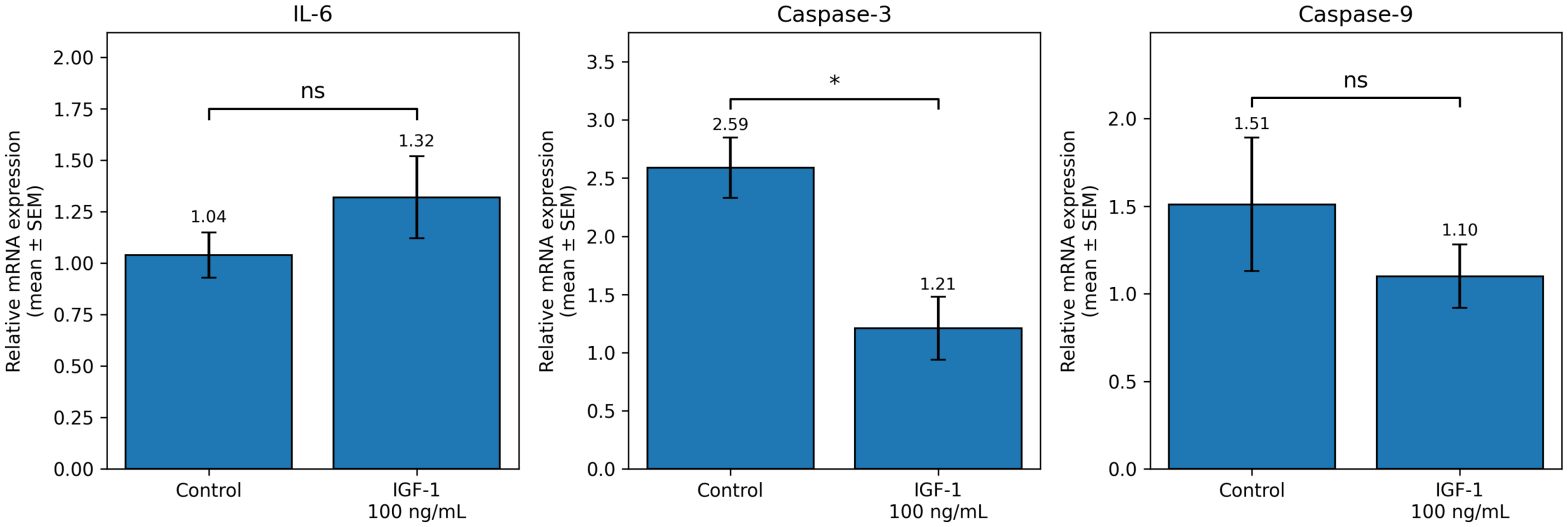
Effect of the optimal IGF-1 dose on apoptosis- and inflammation-related gene expression. Relative mRNA expression of IL-6, caspase-3, and caspase-9 in control (0 ng/mL) and IGF-1 (100 ng/mL) groups. Data are presented as mean ± SEM (*n* = 8 biological replicates). Asterisks indicate significant differences between groups (independent samples *t*-test; *p* < 0.05).

IGF-1 supplementation at 100 ng/mL significantly influenced apoptosis-related gene expression (Table 4; Figure 4). Caspase-3 expression was significantly decreased in the IGF-1 group (1.21 ± 0.27) compared with control (2.59 ± 0.26) (*p* < 0.05). In contrast, IL-6 and caspase-9 expression did not differ significantly between groups (*p* > 0.05), indicating that IGF-1 primarily attenuated the execution-phase apoptotic marker caspase-3 under the present conditions.

**Table 4:** Effect of IGF-1 supplementation on apoptosis-related gene expression.

Treatment groups	*n*	IL-6	Caspase 3	Caspase 9
Control	8	1.04^a^ ± 0.11	2.59^a^ ± 0.26	1.51^a^ ± 0.38
IGF-1 100 ng/mL	8	1.32^a^ ± 0.20	1.21^b^ ± 0.27	1.10^a^ ± 0.18

Relative mRNA expression of IL-6, caspase-3, and caspase-9 in control (0 ng/mL) and IGF-1 (100 ng/mL) groups. Data are presented as mean ± SEM; *n* = 8 biological replicates. Different superscripts within the same column indicate significant differences between groups (independent samples *t*-test; *p* < 0.05).

### 3.4 Correlation analysis among maturation rate and molecular parameters

**Figure 5:**
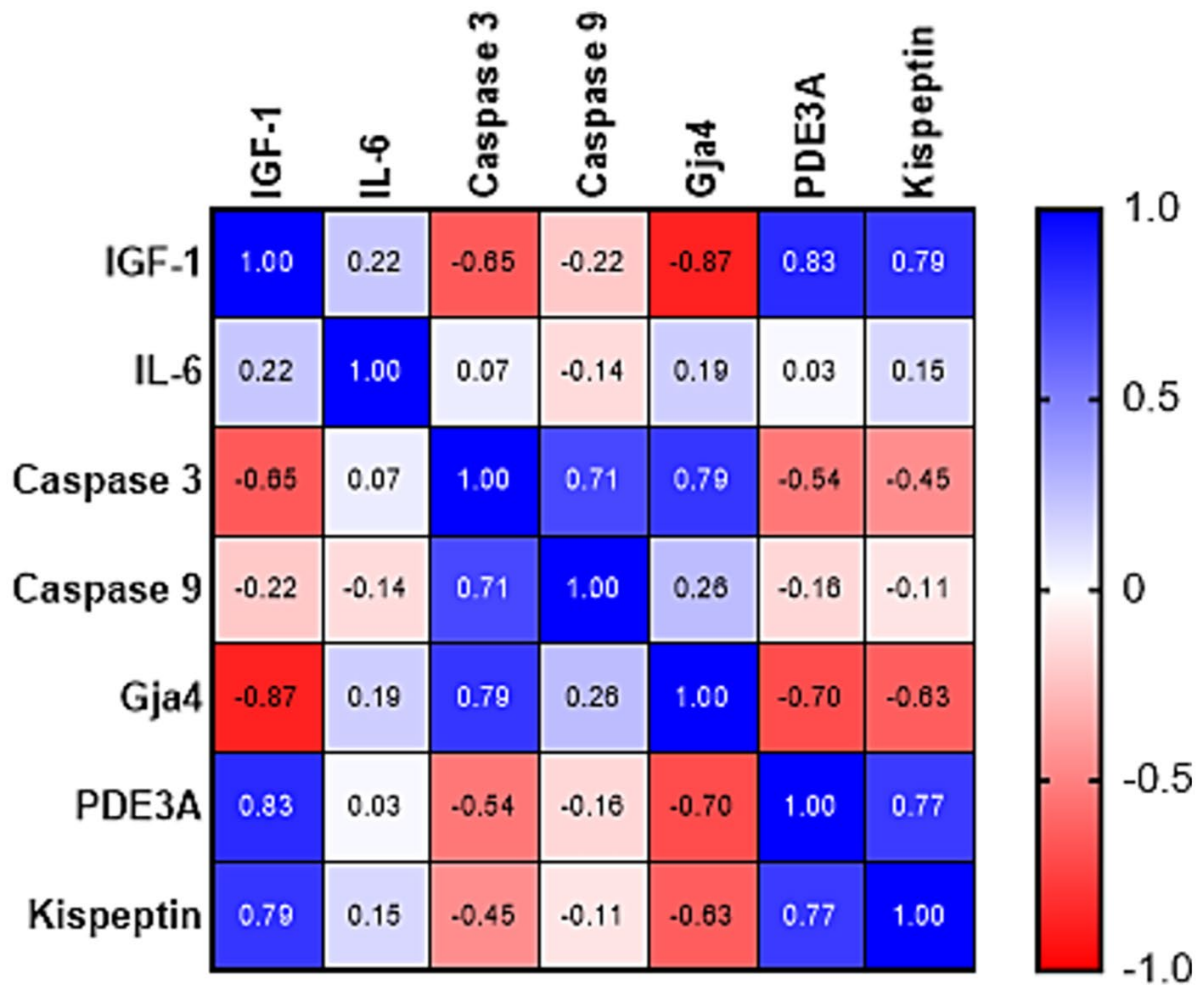
Correlation matrix among maturation outcome and molecular parameters. Pearson correlation matrix showing relationships among maturation rate, GJA4, PDE3A, IL-6, caspase-3, caspase-9, and kisspeptin. Correlation coefficients (*r*) indicate direction and strength of associations. Statistical significance was set at *p* < 0.05.

Pearson correlation analysis revealed clear relationships between maturation outcome and key molecular markers (Figure 5). PDE3A expression showed a strong positive correlation with maturation rate (r > 0.60), while kisspeptin demonstrated a moderate-to-strong positive correlation (r approximately 0.50–0.70). In contrast, caspase-3 expression displayed a strong negative correlation with maturation rate (r < −0.60), and Gja4 expression showed a moderate negative correlation (r approximately −0.40 to −0.60). IL-6 and caspase-9 exhibited weak or non-significant correlations (|r| < 0.40). Inter-parameter relationships further supported coordinated regulation: PDE3A correlated negatively with caspase-3 and Gja4, and caspase-3 correlated positively with caspase-9.

### 3.5 Structural path analysis of IGF-1 effects on maturation rate

**Figure 6:**
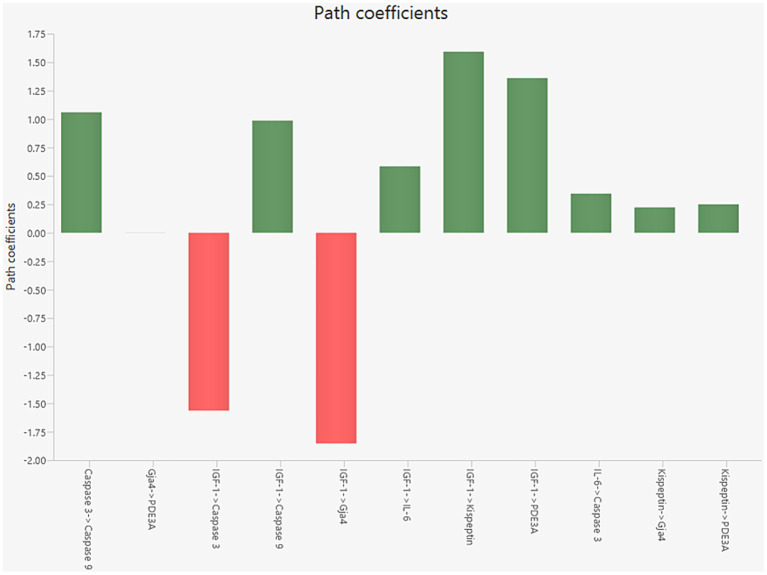
Structural path analysis of IGF-1 effects on maturation rate. Structural path model illustrating direct and indirect effects of IGF-1 supplementation on maturation rate through modulation of Gja4, PDE3A, apoptosis-related genes (IL-6, caspase-3, caspase-9), and kisspeptin. Values on paths represent standardized coefficients (*β*); positive coefficients indicate positive effects and negative coefficients indicate inhibitory effects (significance criteria as defined in the analysis; *p* < 0.05).

**Figure 7:**
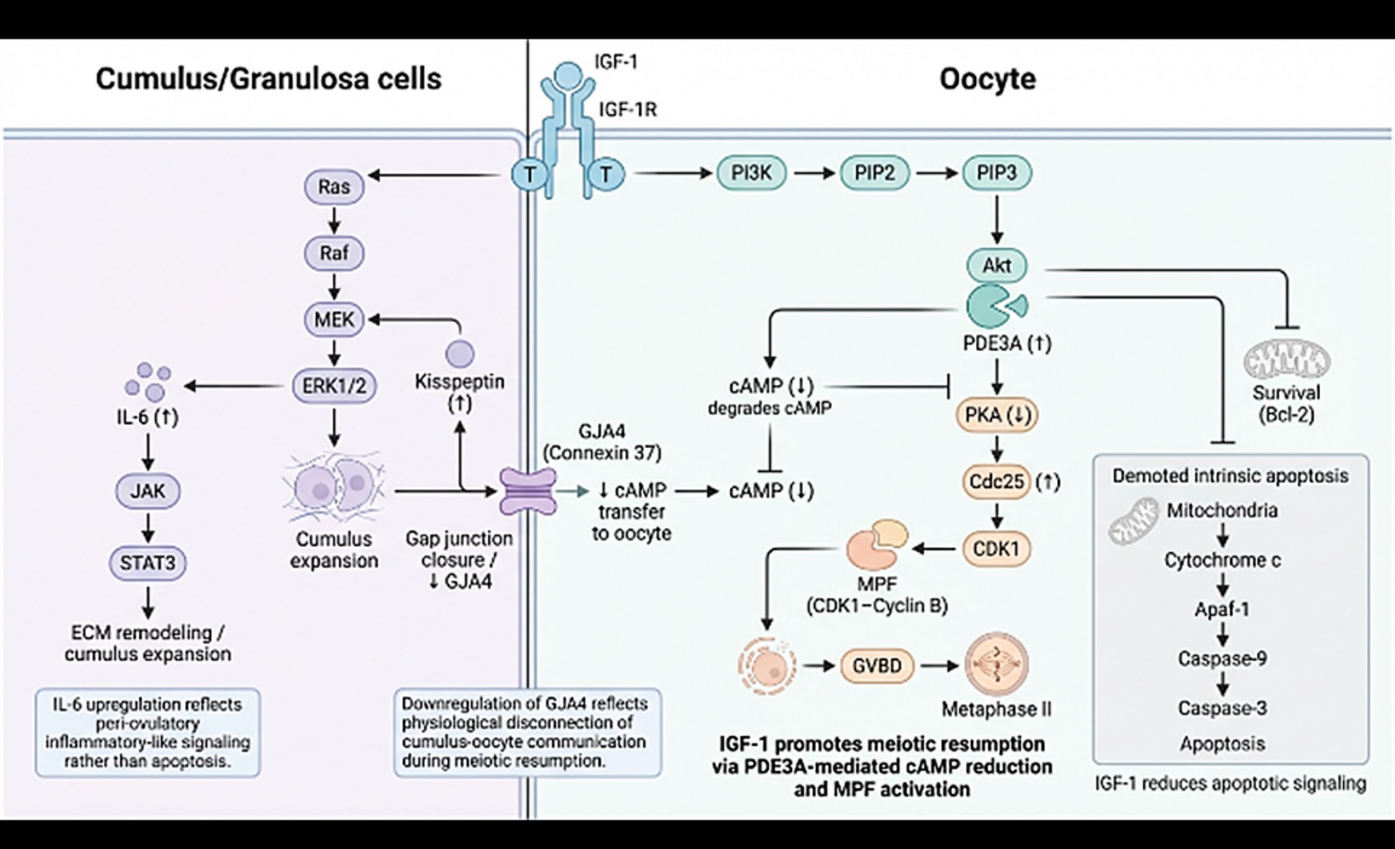
Proposed molecular mechanism of IGF-1–enhanced oocyte maturation in *Capra hircus*. IGF-1 binding to IGF-1R is hypothesized to activate intracellular signaling (e.g., PI3K/Akt), leading to PDE3A upregulation, reduction of intra-oocyte cAMP, activation of MPF (CDK1–Cyclin B), and progression to metaphase II. IGF-1 is also associated with gap junction remodeling via GJA4 regulation and suppression of apoptotic execution via caspase-3 inhibition. Increased kisspeptin signaling and MAPK pathway activity in cumulus cells may further support cumulus function and oocyte competence. Pathway components such as PI3K, Akt, PKA, and Cdc25 are literature-based and were not directly measured in this study. Arrows indicate stimulatory effects and blunt-ended lines indicate inhibitory effects.

Structural path analysis indicated that improvement in maturation rate was predominantly mediated through PDE3A upregulation and caspase-3 suppression (Figure 6). PDE3A exerted a strong positive direct effect on maturation rate (*β* ≥ 0.50), whereas caspase-3 showed a strong negative direct effect (*β* ≤ −0.50). Kisspeptin contributed a moderate positive direct effect (*β* approximately 0.30–0.50), and Gja4 showed a moderate negative direct effect. IL-6 and caspase-9 displayed small or non-significant direct effects. IGF-1 increased maturation rate through indirect pathways involving stimulation of PDE3A and inhibition of caspase-3, supporting a model in which IGF-1 enhances meiotic competence primarily via meiotic activation and reduced apoptotic execution. A conceptual mechanism integrating these interactions is summarized in Figure 7.

## 4 Discussion

This study demonstrates that IGF-1 supplementation enhances the *in vitro* maturation (IVM) of *Capra hircus* oocytes in a dose-dependent manner, with 100 ng/mL identified as the optimal concentration. The observed biphasic response in which 100 ng/mL improved maturation while 150 ng/mL did not provide further benefit supports the concept that IGF-1 acts within an effective concentration window. Comparable dose-dependent effects of IGF-1 have been reported in ruminant and porcine IVM/IVP systems, where moderate supplementation improved outcomes but higher levels did not yield proportional gains (12, 20).

The decline in maturation rate observed at 150 ng/mL suggests a supra-physiological effect of IGF-1, indicating that excessive stimulation may disrupt normal signaling homeostasis. One possible explanation is receptor desensitization or downregulation of IGF-1 receptor (IGF-1R) following sustained high ligand exposure, which has been reported in growth factor signaling systems (21, 22). In addition, excessive activation of downstream pathways such as PI3K/Akt may trigger negative feedback regulators or activate alternative inhibitory signaling cascades, ultimately impairing oocyte competence (22, 26, 27). High IGF-1 concentrations may also alter the balance between survival and stress-related pathways, potentially increasing metabolic burden or oxidative stress within the cumulus–oocyte complex (23).

A central mechanistic finding in the present work is the significant upregulation of PDE3A at the optimal IGF-1 dose, accompanied by a strong positive association with maturation rate. PDE3A is a key regulator of meiotic resumption through hydrolysis of intra-oocyte cAMP, facilitating exit from meiotic arrest and progression to metaphase II (14, 15). The strong path contribution of PDE3A to maturation rate suggests that IGF-1-mediated maturation may be associated with the upregulation of PDE3A-driven meiotic resumption pathways (14). This interpretation is biologically plausible given that IGF-1 signaling can activate intracellular pathways such as PI3K/Akt that support growth and survival processes relevant to oocyte competence (9, 24).

IGF-1 supplementation also significantly decreased Gja4 expression. Connexin 37 (GJA4) mediates gap junction communication between the oocyte and cumulus cells and is important for metabolic coupling during early maturation (13). However, physiological remodeling/closure of gap junctions is considered necessary as oocytes progress toward meiotic competence, because persistent intercellular transfer can maintain inhibitory signals associated with meiotic arrest (13, 21). The negative relationship between Gja4 expression and maturation rate in our dataset is consistent with dynamic connexin regulation described during IVM in other mammalian models (27, 28).

Beyond meiotic activation, IGF-1 improved the apoptotic profile by reducing caspase-3 expression, while caspase-9 and IL-6 remained unchanged. Apoptosis is recognized as a major factor compromising oocyte and embryo competence in animal reproductive biotechnology (16). Specifically, executioner caspases such as caspase-3 are closely linked to decreased developmental potential under *in vitro* stress (17). The strong negative association and direct path effect between caspase-3 and maturation rate in this study underscore that limiting apoptotic execution is an important mechanism supporting maturation competence (16, 17). IGF-1 is also well established as a survival factor, frequently associated with anti-apoptotic signaling through Akt-dependent mechanisms (24). Similar reductions in apoptotic signatures after growth factor supplementation during IVM have been reported in domestic species (20, 25).

Interestingly, IGF-1 significantly increased kisspeptin protein levels and kisspeptin showed a positive relationship with maturation rate. Kisspeptin signaling is classically known for hypothalamic regulation of GnRH, yet accumulating evidence indicates potential direct roles within the ovary, including modulation of oocyte maturation and follicular function (18). Recent reviews and clinical/experimental literature further support kisspeptin as a regulator of reproductive maturation processes beyond the central axis (19). In this context, the present findings suggest that IGF-1 may strengthen intra-ovarian signaling networks that support oocyte competence, with kisspeptin acting as an additional supportive component alongside PDE3A and apoptosis regulation (18, 19).

The correlation matrix and structural path analysis together provide a coherent mechanistic narrative. Correlation analysis indicated positive associations of maturation rate with PDE3A and kisspeptin, and negative associations with caspase-3 and Gja4; the path model further supported directional contributions, identifying PDE3A upregulation and caspase-3 suppression as the most influential determinants of maturation improvement. The integrated interpretation is that IGF-1 enhances meiotic competence mainly by accelerating meiotic resumption via PDE3A–cAMP regulation and by protecting oocytes from apoptotic execution (14, 15). Concurrently, reduced Gja4 expression supports gap junction remodeling appropriate for meiotic progression (13). Kisspeptin elevation may provide additional support to maturation competence through local reproductive signaling (18).

Several limitations should be considered. First, ovaries were obtained from abattoir specimens and estrous cycle stages were not determined, which may introduce biological variability. Second, the study focused on nuclear maturation (MII) and selected molecular markers; although nuclear maturation was significantly improved, future studies are required to confirm if this molecular optimization translates into increased blastocyst yields and high-quality embryo development. Third, the molecular assessment targeted a defined marker set; broader transcriptomic or proteomic profiling could provide deeper mechanistic resolution. Finally, *in vitro* conditions cannot fully replicate the endocrine and paracrine milieu of the *in vivo* follicle.

Despite these limitations, the findings have practical implications for improving ART in small ruminants. Optimizing IVM media with an effective IGF-1 dose can enhance the developmental competence of oocytes recovered from abattoir-derived ovaries, improving IVP efficiency and supporting germplasm preservation for indigenous goat breeds. Such improvements align with Sustainable Development Goal 2 (Zero Hunger) by strengthening sustainable livestock productivity and supporting animal-source food availability, particularly in smallholder production systems.

## 5 Conclusion

IGF-1 supplementation at an optimal dose of 100 ng/mL significantly improves the *in vitro* maturation (IVM) outcome of *Capra hircus* oocytes. The improvement is primarily driven by PDE3A upregulation, consistent with enhanced meiotic resumption through intra-oocyte cAMP regulation and MPF activation, together with reduced caspase-3 expression, indicating attenuation of apoptotic execution during IVM. The concurrent decrease in Gja4 suggests physiological remodeling of cumulus–oocyte gap junction communication accompanying meiotic progression, while increased kisspeptin levels support a contributory role of intra-ovarian reproductive signaling. Collectively, these findings provide a mechanistic basis for optimizing goat IVM systems using IGF-1 to improve oocyte competence. This protocol refinement may enhance IVP efficiency and facilitate germplasm conservation and genetic improvement programs in indigenous small ruminants, supporting Sustainable Development Goal 2 (Zero Hunger) through more sustainable livestock productivity and food security.

## Data Availability

The original contributions presented in the study are included in the article/supplementary material, further inquiries can be directed to the corresponding author.
