# Correction: IGF-1 supplementation improves *in vitro* maturation quality of *Capra hircus* oocytes via PDE3A activation, gap junction remodeling, apoptosis suppression, and kisspeptin signaling

**DOI:** 10.3389/fvets.2026.1897588

**Published:** 2026-06-29

**Authors:** Widjiati Widjiati, Epy Muhammad Luqman, Ninik Darsini, Aulanni'am Aulanni'am, Viski Fitri Hendrawan, Devia Yoanita Kurniawati, Wan Nor Fitri Bin Wan Jaafar

**Affiliations:** 1 Department of Veterinary Anatomy, Faculty of Veterinary Medicine, Universitas Airlangga, Surabaya, Indonesia; 2 Department of Medical Biology, Faculty of Medicine, Universitas Airlangga, Surabaya, Indonesia; 3 Department of Biochemistry, Faculty of Veterinary Medicine, Universitas Brawijaya, Malang, Indonesia; 4 Department of Veterinary Reproduction, Faculty of Veterinary Medicine, Universitas Brawijaya, Malang, Indonesia; 5 Doctoral Program of Veterinary Sciences, Faculty of Veterinary Medicine, Universitas Airlangga, Surabaya, Indonesia; 6 Department of Farm and Exotic Animal Medicine and Surgery, Faculty of Veterinary Medicine, Universiti Putra Malaysia, Serdang, Selangor, Malaysia

**Keywords:** apoptosis, gap junction, IGF-1, *in vitro* maturation, kisspeptin, oocyte, PDE3A, Zero Hunger

Funding information from the Indonesian Endowment Fund for Education (LPDP), on behalf of the Indonesian Ministry of Higher Education, Science and Technology, managed under the EQUITY Program, was erroneously omitted from the **Funding** statement. The correct **Funding** statement reads:

“The author(s) declared that financial support was received for this work and/or its publication. This study was supported by Universitas Airlangga through the Airlangga Research Fund, Airlangga Flagship Research Scheme/Penelitian Unggulan Airlangga 2024 (Contract No. 358/UN3.LPPM/PT.01.03/2024). This paper is a part of activities funded by the Indonesian Endowment Fund for Education (LPDP) on behalf of the Indonesian Ministry of Higher Education, Science and Technology and managed under the EQUITY Program (Contract No. 4300/B3/DT.03.08/2025 and 297/UN3/HK.07.00/2025).”

In the **Acknowledgments**, the required **Acknowledgments** statements for the Airlangga Research Fund and the LPDP-funded EQUITY Program were omitted. The corrected **Acknowledgments** section should read:

“We acknowledge the technical assistance provided by the staff involved in sample collection and laboratory support. The authors also acknowledge Universitas Airlangga through the Airlangga Research Fund, Airlangga Flagship Research Scheme/Penelitian Unggulan Airlangga 2024 (Contract No. 358/UN3.LPPM/PT.01.03/2024), and the Indonesian Endowment Fund for Education (LPDP) on behalf of the Indonesian Ministry of Higher Education, Science and Technology, managed under the EQUITY Program (Contract No. 4300/B3/DT.03.08/2025 and 297/UN3/HK.07.00/2025), for supporting this research activity.”

The original version of this article has been updated.

